# Longitudinal prospective cohort study to assess peripheral motor function with extensive electrophysiological techniques in patients with Spinal Muscular Atrophy (SMA): the SMA Motor Map protocol

**DOI:** 10.1186/s12883-023-03207-5

**Published:** 2023-04-24

**Authors:** Leandra A. A. Ros, H. Stephan Goedee, Hessel Franssen, Fay-Lynn Asselman, Bart Bartels, Inge Cuppen, Ruben P. A. van Eijk, Boudewijn T. H. M. Sleutjes, W. Ludo van der Pol, Renske I. Wadman

**Affiliations:** 1 Department of Neurology & Neurosurgery, University Medical Center Utrecht, UMC Utrecht Brain Center, Utrecht University, Heidelberglaan 100, 3508 GA Utrecht, The Netherlands; 2 Child Development and Exercise Centre, Wilhelmina Children’s Hospital, University Medical Center Utrecht, Utrecht University, Utrecht, The Netherlands; 3 Biostatistics and Research Support, Julius Center for Health Sciences and Primary Care, University Medical Center Utrecht, Utrecht University, Utrecht, The Netherlands

**Keywords:** Spinal muscular atrophy, Nusinersen, Risdiplam, Electrophysiological techniques, Motor unit

## Abstract

**Background:**

Hereditary spinal muscular atrophy (SMA) is a motor neuron disorder with a wide range in severity in children and adults. Two therapies that alter splicing of the *Survival Motor Neuron 2 (SMN2)* gene, i.e. nusinersen and risdiplam, improve motor function in SMA, but treatment effects vary. Experimental studies indicate that motor unit dysfunction encompasses multiple features, including abnormal function of the motor neuron, axon, neuromuscular junction and muscle fibres. The relative contributions of dysfunction of different parts of the motor unit to the clinical phenotype are unknown. Predictive biomarkers for clinical efficacy are currently lacking. The goals of this project are to study the association of electrophysiological abnormalities of the peripheral motor system in relation to 1) SMA clinical phenotypes and 2) treatment response in patients treated with *SMN2*-splicing modifiers (nusinersen or risdiplam).

**Methods:**

We designed an investigator-initiated, monocentre, longitudinal cohort study using electrophysiological techniques (‘the SMA Motor Map’) in Dutch children (≥ 12 years) and adults with SMA types 1–4. The protocol includes the compound muscle action potential scan, nerve excitability testing and repetitive nerve stimulation test, executed unilaterally at the median nerve. Part one cross-sectionally assesses the association of electrophysiological abnormalities in relation to SMA clinical phenotypes in treatment-naïve patients. Part two investigates the predictive value of electrophysiological changes at two-months treatment for a positive clinical motor response after one-year treatment with *SMN2*-splicing modifiers. We will include 100 patients in each part of the study.

**Discussion:**

This study will provide important information on the pathophysiology of the peripheral motor system of treatment-naïve patients with SMA through electrophysiological techniques. More importantly, the longitudinal analysis in patients on *SMN2*-splicing modifying therapies (i.e. nusinersen and risdiplam) intents to develop non-invasive electrophysiological biomarkers for treatment response in order to improve (individualized) treatment decisions.

**Trial registration:**

NL72562.041.20 (registered at https://www.toetsingonline.nl. 26–03-2020).

## Background

Spinal muscular atrophy (SMA) is an important genetic cause of mortality in infants and progressive motor impairment in children and adults [1, 2]. SMA is caused by a loss of function of the *Survival Motor Neuron 1* (*SMN1)* gene on chromosome 5q, which leads to degeneration of alpha motor neurons in the anterior horn of the spinal cord and structural and functional changes in axons, neuromuscular junctions (NMJ) and muscle fibres [2–9]. SMA severity ranges from onset in infancy to adulthood and inversely correlates with *SMN2* copy number. The clinical classification system distinguishes SMA types 1–4, in descending levels of severity. Infantile onset SMA type 1 has, when left untreated, a life-expectancy < 24 months, while late-infantile and childhood onset SMA types 2 (‘sitters’) and 3 (‘walkers’) are characterized by severe disability. Adult-onset SMA type 4 is characterized by relatively mild proximal muscle weakness. The natural history of SMA is one of progressive muscle weakness, irrespective of SMA type [10–14]. Two therapies that alter *SMN2*-splicing, i.e. ‘nusinersen’ (Spinraza) and ‘risdiplam’ (Evrysdi), can improve motor function in children and adults [15–18]. Nusinersen is an intrathecally administered antisense oligonucleotide (ASO) that supposedly exerts its effects on alpha-motor neurons [19], while risdiplam is an orally supplied drug with potentially more systemic effects [20]. Treatment inefficacy (i.e. inability to stabilize motor function) may only become apparent after years of treatment due to the insensitivity of available clinical scores to detect motor decline within this time frame. The burden of treatment and the high costs require the development of more sensitive tools that enable clinical response at an early stage.

Experimental, pathological and clinical studies have shown that dysfunction of several parts of the motor unit, i.e. soma, axon and NMJ may underlie motor symptoms in SMA. It is not known how dysfunction of the constituting parts of the motor unit contribute to weakness or how treatment improves motor unit function.

Electrophysiological techniques, in particular combinations of the compound muscle action potential (CMAP) amplitude [21, 22], motor unit number estimation (MUNE) [21, 23–25], excitability testing [26, 27] and repetitive nerve stimulation [3, 28], are promising tools to characterize the constituting parts of the motor unit in patients with SMA in more detail and may be useful as biomarkers for response to treatment [24, 25, 27]. However, these techniques have never been studied combined in large patient cohorts. We therefore aim to use an integrated set of non-invasive electrophysiological techniques, which we coined the “SMA Motor Map”, to evaluate the pathophysiology of the peripheral motor system in SMA and alterations in its function after the start of treatment.

## Methods

### Study setting and design

We conduct this investigator-initiated, monocentre, longitudinal cohort study at the Netherlands SMA Center of the University Medical Center Utrecht, a tertiary referral centre for neuromuscular diseases in the Netherlands and the only designated treatment centre for SMA in the Netherlands.

**Fig. 1:**
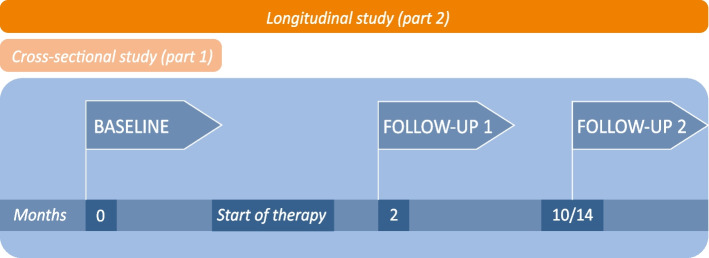
Study protocol of the SMA Motor Map. Part one is performed in *SMN2*-splicing modifier treatment-naïve patients (baseline). If the participant starts treatment with either nusinersen or risdiplam and consents to participate longitudinally, the participant will be included in part two (additional measurement at two months and 10 to 14 months, depending on therapy). Participants can take part at part one without participation in part two. The study protocol includes electrophysiological assessments (compound muscle action potential (CMAP), CMAP scan, motor nerve excitability testing, repetitive nerve stimulation (RNS) and the sensory nerve action potential (SNAP)) at all visits. Functional motor assessments (Hammersmith Functional Motor Scale Expanded (HFMSE), Revised Upper Limb Module (RULM) and Adult Test of Neuromuscular Disorders (ATEND)) are performed at baseline and at the second follow-up measurement. The electrophysiological test set is performed in the same order at all visits

The study consists of two parts, a cross-sectional baseline part (part one) and a consecutive longitudinal follow-up (part two) including assessments at 2 months and approximately after one year (Fig. 1). Participants can take part at part one without participation in part two.

The study schedule is presented in Table 1. At baseline (part one), we investigate the integrity and (dys)function of the various parts of the peripheral motor system across the spectrum of SMA. Eligible patients that agree to participate in this study will complete at least one visit (part one), which will be executed when the patient is treatment-naïve. We will enrol 25 age-matched healthy controls as reference in order to confirm that alterations are SMA- and not age-specific of motor unit function. If the participant starts treatment with either nusinersen or risdiplam and consents to participate longitudinally, the analysis of part one will serve as baseline measurement for the longitudinal study (part two). Part two of the study consists of assessments at two months and approximately one year after the start of treatment with either nusinersen or risdiplam. This part of the study allows the evaluation of the biomarker potential of the SMA Motor Map protocol to predict the clinical response of nusinersen and risdiplam [15–18].

**Table 1:**
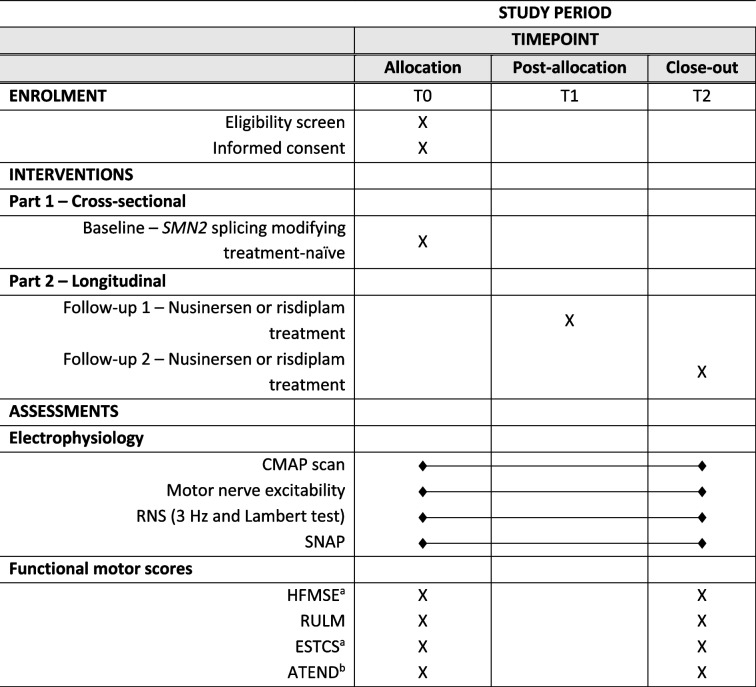
Study schedule of enrolment, interventions and assessments

*ATEND* Adult test of neuromuscular disorders, *CMAP* Compound muscle action potential, *ESTCS* Endurance shuttle combined score, HFMSE Hammersmith functional motor scale expanded, *RNS* Repetitive nerve stimulation, *RULM* Revised upper limb module, *SMN2* *Survival motor neuron 2*gene, *SNAP* Sensory nerve action potential, *T0* Baseline measurement, *T1* Two months after start of treatment, *T2* Fourteen or ten months after start of treatment with nusinersen or risdiplam, respectively

^a^Assessments only for patients treated with nusinersen

^b^Assessment only for patients treated with risdiplam

The first participant was included on 25 May 2020. Study completion is in the first quartile of 2023.

### Participants

#### Recruitment

We screened all Dutch patients aged ≥ 12 years registered in our national SMA registry to start treatment with *SMN2*-splicing modifying therapies (nusinersen or risdiplam) according to the Dutch reimbursement conditions and informed them about our study. Conditional reimbursement for treatment with nusinersen in the Netherlands started in January 2020. One of the conditions for reimbursement of nusinersen is compulsory clinical and physiotherapeutic assessments at baseline, after two months and followed by every four months. Nusinersen is dosed according to the manufacturer’s schedule [15, 16]. Risdiplam became available in 2021 through a compassionate use program for patients with SMA types 1 and 2, who are not eligible for treatment with nusinersen. Risdiplam is orally administered at a daily dose of 5 mg [17, 18].

All patients are monitored for safety and efficacy assessments at baseline, after two months and then every eight months.

We will recruit 25 age-matched healthy controls through our website (www.smaonderzoek.nl), the newsletter for patients with SMA and the newsletter of the patient organization Spierziekten Nederland.

#### Eligibility criteria

The details of the inclusion and exclusion criteria are provided in Table 2.

**Table 2:** Inclusion and exclusion criteria SMA Motor Map

Inclusion criteria	Loss of function of the *SMN1* gene	
	Clinical diagnosis of SMA types 1–4	Type 1: never able to sit independently
		Type 2: achieved the ability to sit independently, but never able to walk independently
		Type 3: achieved the ability to walk independently and age at onset before 18 years
		Type 4: achieved the ability to walk independently and age at onset ≥ 18 years
	Age 12 years or older	
	Treatment-naïve for *SMN2-* splicing modifying drugs	
	Given oral and written informed consent by the patient, and their parents or legal representative in case of minors^a^	
Exclusion criteria	Definite	Strong apprehension against the performance of electrophysiological techniques of any kind
	Relative	Use of pyridostigmine^b^
		Use of medication affecting peripheral nerve ion-channel currents^c^

^a^Minors are participants aged 12 through 16 years old as defined by the regulation of the Dutch Central Committee on Research Involving Human Subjects

^b^In case of pyridostigmine use by indication of SMA, patients are asked to stop treatment one day prior to examination

^c^These include: transient and persistent Na-currents which are mediated by Nav1.6 (SCN8A) ion-channels, fast potassium currents which are mediated by Kv1.1 (KCNA) ion-channels, slow potassium currents which are mediated by KCNQ1 ion-channels, and hyperpolarization-activated inward cation currents which are mediated by HCN-channels. Medication that is not allowed include quinidine, lidocaine, amiodarone, sotalol, amitriptyline, carbamazepine; though the list is not complete and in each case the drug administered by the patient will be checked for its influence on the above ion-currents

#### Sample size calculation

We conducted a sample size calculation for the two parts of the study separately.

We based our power analysis for the cross-sectional study (part one) on the axonal excitability technique [26]. Based on a previous study [26], we will need to include 25 patients per SMA type in order to detect a difference between the four SMA types and reach a power of 90% (two-sample t-test power calculation; α = 0.05, delta = 0.1, sd = 0.1) [26].

For the analysis of the SMA Motor Map predictive biomarker potential, our power analysis is based on a range of expectations. The response rate on nerve conduction techniques after two months is currently unknown and so is the predictive value of the SMA Motor Map to predict a clinical response after one year. We used a simulation approach to estimate the empirical power for a range of scenarios using a fixed sample size of 100 patients. In previous trials, the clinical response rate in children on the Hammersmith Functional Motor Scale Expanded (HFMSE) was 60% [15, 16]. To be conservative, we assumed a clinical response rate of 50%. We evaluated the following scenarios: response rate of the targeted SMA Motor Map ranging from 10 to 60%, and the predictive value of the electrophysiological techniques ranging from 50% (the same as flipping a coin) to 80%. We simulated data under a logistic model and counted the number of times the *p*-value for the coefficient of electrophysiology was less than 0.05 in 10,000 simulations (for each scenario). Based on these analyses, a sample size of 100 patients provides at least 80% power if the predictive value of electrophysiology is at least 75% and the clinical response rate at least 50%. The simulation script is available on request.

### Data collection

#### Outcomes

##### Electrophysiological techniques

We created a protocol consisting of multiple non-invasive peripheral electrophysiological techniques applied on the median nerve unilaterally. This protocol comprises the following techniques and is executed in the same subsequent order in all patients at all visits: 1) compound muscle action potential (CMAP) scan, 2) motor nerve excitability testing, 3) repetitive nerve stimulation (RNS) and 4) the sensory nerve action potential (SNAP) [3, 21, 25, 27].

We perform all tests on the patient’s dominant hand. If for any reason investigation at this side is precluded (e.g. because of severe contractures), all analyses are done on the non-dominant side.

Nerve and muscle temperature in the forearm is brought to 37 °C degrees by wrapping the arm in a warm water blanket through which water flows constantly at 37 °C degrees (Cincinnati Sub-zero Norm-O-Temp with a Cincinnati Plastipad infant blanket) for 30 min before testing. During testing the temperature is maintained at 37 °C degrees by the same procedure [29].

We perform testing with patients in either sitting (wheelchair bound patients) or supine position (ambulant patients). Patients are instructed to rest their arm in a natural position, ensuring muscle relaxation as much as possible to prevent movement or (postural) tremor. Follow-up analysis is performed with the arm and hand in the same position as during baseline measurements. We use photographs of the position of the arm and hand to ensure the same position at follow-up.

We use an isolated bipolar constant current stimulator (DS5, Digitimer, UK) and amplifier (D440-4, Digitimer, UK) in all recordings. All are connected to the Qtrac-S software (Institute of Neurology, Queen Square, London, UK) controlling the measurements and acquisition using a data acquisition device (PCI-6221, National Instruments), with sampling at 10 kHz.

We measure the thenar CMAPs for motor recordings (CMAP scan, motor nerve excitability and RNS) by 1 cm surface cup electrodes in belly tendon montage and stimuli are applied with the cathode at the level of the wrist at 7 cm from the active recording surface electrode and the anode 10 cm proximal over the radial side of the arm (3M Red Dot electrodes). Optimal placement of the cathode at the level of the wrist is manually determined with a stimulation pen (Motor Point Pen, Compex, Switzerland). Signals are amplified by a factor of 300 for motor recordings with filter settings of 10 Hz to 10 kHz.

We perform one sensory assessment (maximum SNAP) of the median nerve. Stimuli (0.5 ms duration) are given at 12 cm from the active ring electrodes, which are positioned around the proximal and distal interphalangeal joints of the third digit. The stimuli are applied with the electrodes at the same position as the motor nerve recordings. Signals are amplified by a factor of 10,000 with filter settings of 10 Hz to 3 kHz.

All electrodiagnostic tests are performed by the same investigator (LR), who is trained to perform these assessments. If this investigator is unavailable, the measurements will be performed by another trained study group member (BS).

##### Compound Maximum Action Potential (CMAP)

We will obtain the maximum CMAP amplitude from the CMAP scan, which is a basic parameter for the analysis of motor axon integrity. Previous studies have correlated active disease course with a dramatic fall of the CMAP amplitude as a reflection of severe motor neuron loss [21, 22].

##### CMAP scan

The CMAP scan reflects the gradual recruitment of the motor unit pool that innervates the investigated muscle [30, 31]. Motor unit analysis by CMAP scans in patients with SMA has shown differences between SMA types and response to treatment by means of motor unit size and number [21, 32]. The CMAP scan generally will take less than 10 min to perform. For CMAP scan analysis we use the MScan-application, in which the maximum CMAP is manually determined after which approximately 500 – 700 automated stimuli are applied (at 2 Hz, 0.1 ms stimulus duration) with exponential decreasing currents until the lowest-threshold motor unit becomes subthreshold [33, 34].

##### Motor nerve excitability testing

Motor nerve excitability testing is a non-invasive method that assesses changes in resting membrane potential, ion channel function and nodal and internodal membrane properties (TRONDNF protocol, version 25/1/2019). Analysis of motor nerve excitability previously showed altered K + conductance following active axonal loss and a hyperpolarisation of axons in severe SMA, which both responded to nusinersen treatment [26, 27].

Motor nerve excitability testing generally will take 10–15 min to perform. The motor nerve excitability recordings are initiated by the stimulus response (SR) test (relation between stimulus current and response amplitude) to identify the target response (set at 40% of the maximum CMAP amplitude) for the other four main tests (strength-duration test, threshold electrotonus, current/voltage relationship, and recovery cycle). The combination of these tests provides information on the activity of nodal persistent and transient Na^+^-channels, nodal and internodal slow and fast K^+^-channels, internodal hyperpolarizing-activated cyclic nucleotide-gated (HCN) channels, nodal and internodal leak channels, Barrett and Barrett (i.e., high-resistance pathways through the myelin sheath) conductance, and changes in resting membrane potential. For that, we will apply a well-established mathematical model (Qtrac-P, MEMfit tool) of a single axon to help identify the most likely dysfunctional mechanism that can explain the observed changes in axonal excitability [35, 36].

##### Repetitive nerve stimulation

RNS is used to assess post- and presynaptic NMJ signal transmission, and generally will take 10 min to perform. NMJ signal transmission has been assessed in patients with SMA, showing 49%-60% of patients having a decremental response as a sign of post-synaptic dysfunction [3, 28].

For post-synaptic analysis, a train of 10 supramaximal consecutive stimuli is given at 3 Hz with 0.1 ms stimulus duration [3]. Stimulus intensity is individually set on supramaximal level (approximately 10% above the intensity required for a maximum CMAP response). For pre-synaptic analysis, we use the Lambert test. The intensity for supramaximal stimulation is redetermined as described above. Supramaximal stimuli are given prior to and after 10 s of maximum voluntary contraction (MVC) by isometric contraction of the thenar muscles by pushing the thumb against a fixed surface/object.

In both post- and presynaptic analyses, the CMAP response of consecutive stimuli is used to analyse the presence and size of decremental or incremental responses. A 10% decrement is specific for primary post-synaptic transmission dysfunction [37]. A 60% or more increment is specific for pre-synaptic transmission dysfunction [38].

##### Sensory nerve action potential

The SNAP assesses the afferent part of the median nerve. Although patients with SMA generally do not have sensory symptoms, alterations in the sensory nerves and circuit were reported in animal and human studies [39–42]. We added the maximum SNAP analysis to assess afferent components of the median nerve, in addition to the efferent motor system. For the SNAP analysis we give supramaximal stimuli to record three consecutive maximum SNAPs (median nerve, third digit). From these three recorded maximum SNAPs we determine the mean to use for further analyses.

#### Clinical assessments

We collect and analyse patient characteristics (e.g., age at onset, *SMN2* copy number, disease duration, use of concomitant medication and comorbidities). Patient characteristic have been longitudinally collected in the SMA registry from 2010 onwards and are reassessed at follow-up.

Clinical parameters to assess motor function, arm function and fatigability in patients are collected from the database as they are performed to assess efficacy of the current *SMN2*-splicing modifying treatment. Motor function assessments include Hammersmith Functional Motor Scale Expanded (HFMSE) [43–45], Adult Test of Neuromuscular Disorders (ATEND) [46], Revised Upper Limb Module (RULM) [47] and Endurance Shuttle Combined Score (ESTCS) [48]. These functional motor scales are validated for SMA patients to test different motor abilities. None of the scales reflect the whole disease spectrum of SMA and have either ceiling or floor effects for the most severe or milder affected patients [11, 49].

Treatment protocols for risdiplam or nusinersen are under conditional reimbursement in the Netherlands and included standardized motor assessments. All patients are assessed using the RULM.

Patients treated with nusinersen are additionally assessed with HFMSE and ESTCS. The ESTCS is performed according to the highest level of motor function: patients with hand and forearm function perform the Endurance Shuttle - Nine Hole Peg Test (ESNHPT), patients who are able to lift their arm against gravity perform the Endurance Shuttle - Box and Block Test (ESBBT) and patients who are able walk independently perform the Endurance Shuttle - Walk Test (ESWT) [48]. Patients treated with risdiplam are assessed with the ATEND next to the RULM.

In case the last recorded score in the registry at baseline assessment is more than 18 months old, these scores will be reassessed as part of the study protocol.

#### Adverse events

All adverse events (AEs) occurring during the test visits, either reported spontaneously by the participant or observed by the investigator or study staff members are recorded and if necessary, appropriate measures are taken.

### Statistical analysis

In part one of this study, we will assess the association between clinical severity (e.g. SMA type) and electrophysiological state of the peripheral motor system. Correlations between electrophysiological outcomes and clinical variables will be assessed by Spearman’s rho test for continuous variables and the Kruskal Wallis to compare a continuous variable within groups. For comparison of proportions, we will use the chi-square test. We will use a multinomial model to analyse whether the outcomes of the CMAP scan, nerve excitability tests and/or RNS show differences between SMA types. Missing data in baseline characteristics will be addressed by creating multiple imputed datasets using predictive mean matching. Results across imputations will be pooled using Rubin’s rules.

In part two of this study, we will analyse the predictive value of the SMA Motor Map at two months by means of the clinical effects and its correlation with the SMA Motor Map compartments at approximately one year using a logistic model. The predictive value of the SMA Motor Map for clinical response is based upon the following definitions:

- Clinical response is defined as stabilization or improvement on the clinical score (including RULM and/or HMFSE) over a period of approximately one year.
- The primary SMA Motor Map response is defined as stabilization or improvement of the motor unit number at the CMAP scan. The secondary SMA Motor Map response is defined as stabilization or improvement of any of the other electrophysiological markers derived from the advanced electrophysiological techniques (e.g. presence of decremental response, CMAP max, excitability tests).

We will use a ROC curve to determine the additive value of the SMA Motor Map (e.g. motor unit number) at two months to predict clinical response at one year. The comparative logistic model will consist of only information collected at baseline (i.e. month 0).

### Data managment

The following measures will be taken to assure the confidentiality and anonymity of the participants’ data or documents collected in the SMA database on the secured UMC Utrecht drivers (which also provides automated and regular back-up of the data): a) each participant will be identified in an electronic database by a unique seven digit code; b) the list of participant names corresponding to the codes will be stored in a separate encrypted electronic database, safeguarded by the principal investigator; c) only study investigators will have access to the databases and examine individual data or documents; d) all logins will be recorded; e) adopt strict precautions to prevent access to the data or documents by non-authorised persons; f) the handling of data and documents will comply with the General data protection regulation (in Dutch: Algemene Verordening Gegevensbescherming (AVG)).

### Ethics, dissemination and safety monitoring

This study was approved by the Medical Ethics Committee of the University Medical Center Utrecht (No. 20–143 (Version 3)/NL72562.041.20) and registered in the Dutch registry for clinical studies and trials (https://www.toetsingonline.nl). The trial is monitored by an internal party of the University Medical Center Utrecht.

We follow standard procedures to obtain oral and written consent from all participants and/or their parents or legal representative in case of minors. Minors are participants aged 12 through 16 years old as defined by the regulation of the Dutch Central Committee on Research Involving Human Subjects. The study is conducted according to the principles of the Declaration of Helsinki, adapted 19–10-2013, and in accordance with the Medical Research Involving Human Subjects Act (WMO). The code of Conduct as agreed upon 2001 by the Dutch organization of Pediatrics will be used. The study is partly done in minors, which means that in any case of resistance the test and research protocol will be terminated. Resistance means that the participant’s behavior obviously differs from or is more excessive compared to participant’s normal behavior. The national rules of the Dutch Association of Pediatrics for protection of minor study participants, are followed during the entire study.

The reporting of this study protocol conforms to the Standard Protocol Items: Recommendations for Interventional Trials (SPIRIT) guidelines [50]. The results of this study will be shared with the academic and medical community by presentations at scientific meetings, as well as publication of article(s) in international, peer-reviewed, open-access journals, funding, and patient organizations in order to contribute to optimization of medical care and quality of life for patients with SMA.

## Discussion

We will conduct a longitudinal cohort study in patients with SMA to analyse the use of an integrated set of well-established and more recently developed electrophysiological techniques – the ‘SMA Motor Map’ – to 1) provide further insight into the pathophysiology of SMA and 2) investigate its value as a biomarker to predict clinical response after treatment with *SMN2*-splicing modifying therapies nusinersen or risdiplam. The unique combination of electrophysiological techniques will allow us to investigate the (dys)function of the whole peripheral motor system and the contribution of the different parts to the clinical phenotype in *SMN2*-splicing modifying treatment-naïve children above 12 years, adolescents, and adults with SMA types 1–4. The electrophysiological techniques are applied on the median nerve unilaterally in a timeframe of one year of treatment, to ensure a feasible and endurable protocol. In addition, we will investigate the value of the SMA Motor Map techniques for predictive purposes in *SMN2-*splicing modifying treatments for treatment response. More importantly, by following patients on these therapies (i.e. nusinersen and risdiplam) over time in the longitudinal study, we aim to develop non-invasive electrophysiological biomarkers in order to improve (individualized) treatment decisions.

## Data Availability

Not applicable.

## Abbreviations

AEs: Adverse events
ASO: Antisense oligonucleotide
ATEND: Adult Test of Neuromuscular Disorders
CMAP: Compound muscle action potential
ESBBT: Endurance Shuttle - Box and Block Test
ESNHPT: Endurance Shuttle - Nine Hole Peg Test
ESTCS: Endurance Shuttle Combined Score
ESWT: Endurance Shuttle - Walk Test
HCN: Hyperpolarizing-activated cyclic nucleotide-gated
HFMSE: Hammersmith Functional Motor Scale Expanded
MUNE: Motor unit number estimation
MVC: Maximum voluntary contraction
NMJ: Neuromuscular junction
RNS: Repetitive nerve stimulation
RULM: Revised Upper Limb Module
SMA: Spinal muscular atrophy
SMN1: Survival motor neuron 1 gene
SMN2: Survival motor neuron 2 gene
SNAP: Sensory nerve action potential
SPIRIT: Standard Protocol Items: Recommendations for Interventional Trials
SR: Stimulus response
